# A Rare Form of Metastatic Melanoma in an HIV-Infected Patient – A Diagnosis to Remember

**DOI:** 10.7759/cureus.20743

**Published:** 2021-12-27

**Authors:** Marta Leal-dos-Santos, Diana Seixas, Emanuel Gouveia, Mariana Cravo, Fernando Maltez

**Affiliations:** 1 Infectious Diseases, Hospital de Curry Cabral - Centro Hospitalar Universitário de Lisboa Central (CHULC), Lisbon, PRT; 2 Oncology, Instituto Português de Oncologia Francisco Gentil, Lisbon, PRT; 3 Dermatology, Instituto Português de Oncologia Francisco Gentil, Lisbon, PRT

**Keywords:** cancer in hiv patients, gastro-intestinal metastasis, malignant melanoma, human immunodeficiency virus infection, metastatic melanoma

## Abstract

Malignant melanoma (MM), which is amongst the rarest skin cancers, still remains one of the deadliest and most likely to spread, and, in human immunodeficiency virus (HIV)-infected patients, generally has a more aggressive behaviour. Although gastrointestinal (GI) tract metastases are frequent, secondary symptomatic colonic disease is rare. We present the case of a 76-year-old HIV-infected patient, with a 15-month history of GI and constitutional symptoms and a previous diagnosis of malignant melanoma. Diagnostic workup revealed metastatic involvement of the cecum. This case highlights the need to bear in mind the metastatic involvement of the GI tract by MM, and MM itself, especially in HIV-infected patients.

## Introduction

Malignant melanoma (MM), an epithelial cancer, arises from melanocytes, which can be found in a variety of tissue types. Despite being the rarest of skin cancers, it remains the deadliest and most likely to spread [1]. In human immunodeficiency virus (HIV)-infected people, melanoma has generally a more aggressive behavior and lower survival compared to uninfected people [2]. Although MM metastasizes frequently to the gastrointestinal (GI) tract, secondary colonic disease is rare [3]. These metastases often present with nonspecific symptoms such as fatigue, weight loss, obstruction, and malabsorption [1,4]. Symptomatic GI involvement is found in less than 5% of melanoma cases [1,4]; however, it has been demonstrated in up to 60% at post-mortem [1].

## Case presentation

We present the case of a 76-year-old man, with a 15-month history of melena, anorexia, tiredness, and 14 kg weight loss. The patient is HIV-positive, since 2013, and has a positive HLA B*5701 and was on tenofovir (TDF), emtricitabine (FTC) and rilpivirine (RPV). He had good therapeutic adherence and immunovirological control, showing long-lasting undetectable viral loads and CD4^+ ^counts above 400 cells/mm^3^. His lowest haemoglobin was 5.8 g/dL, requiring hospital admission and blood transfusions four months into the clinical course. He had a previously excised cutaneous melanoma of the shoulder one year prior. The MM was classified as T4N0Mx, and the sentinel lymph node was negative.

**Figure 1:**
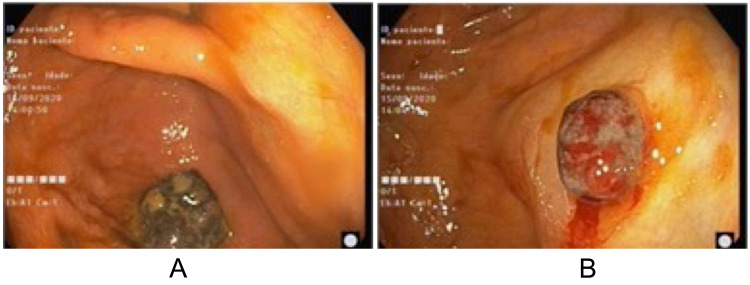
First colonoscopy (A) Angiomatous lesion of the cecum, 20mm, non-detachable with lavage. (B) Angiomatous lesion of the proximal ascending colon, 12mm, non-detachable with lavage.

**Figure 2:**
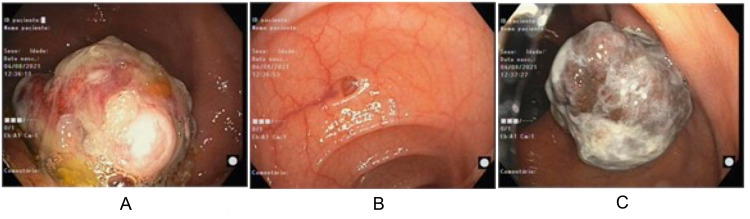
Second colonoscopy (A) Large sessile friable lesion of the cecum, covered in fibrinous exudate and with hyperpigmented areas (4cm). (B) Sigmoid - normal aspect. (C) Semi-pedunculated lesion of the proximal ascending colon, hyperpigmented (3cm).

Initial imaging by computed tomography (CT) demonstrated lipomatous infiltration of the submucosa of the right proximal colon and initial colonoscopy showed two non-detachable angiomatous lesions of 12 and 20mm (Figure 1) that were not biopsied due to bleeding risk. Subsequent colonoscopy showed a cecal 4cm sessile lesion, friable with fibrinous exudate, and a 3cm semi-pedunculated hyperpigmented lesion (Figure 2).

The biopsy result was consistent with malignant melanoma (S100+, Melan-A+, AE1/AE3-). The patient’s clinical course was further complicated by post-colonoscopy hemorrhaging addressed conservatively with blood transfusions. Currently, the patient is under follow-up by the oncology team and is scheduled to have a hemicolectomy with concomitant systemic therapy still under consideration.

With regard to antiretroviral therapy, bearing in mind future drug interactions, we switched the patient from TDF/FTC/RPV to TDF/FTC and doravirine (DOR), with sustained undetectable viral load and good CD4^+^ counts.

## Discussion

It has been estimated that the incidence of melanoma in HIV-infected people is 2.6-fold higher than in uninfected ones [2], and malignant melanoma is the most common carcinoma to metastasize to the GI tract, followed by breast and lung cancer [1]. One of the most common signs of colonic metastasis in patients with melanoma is intestinal bleeding [3] as was the case with our patient. Nevertheless, recent studies show that up to 31% of stage III and stage IV patients could have asymptomatic GI metastasis [1].

Several imaging techniques and endoscopy methods can be used to make a diagnosis [1,4]. We initially addressed the patient by performing a CT scan given it has a sensitivity of 60-70% for the detection of intestinal MM metastases [1,5]. Because of its high sensitivity and specificity subsequent endoscopic studies were performed warranting a diagnosis by showing typical lesions - ulcerated melanotic nodules or mass lesions with necrosis and melanosis [4].

Our patient’s diagnosis was made 17 months after the MM diagnosis which is in line with the previously documented time frame period between the diagnosis of primary MM and the identification of a gastrointestinal metastasis (2 to 180 months) [1]. Studies suggest a high index of suspicion and a low threshold for biopsy in HIV-positive patients regardless of their CD4+ count [2].

Biopsies play an essential role in the diagnosis and identification of the tumor’s origin. It has been shown that metastasectomy in conjunction with systemic therapy is superior to either modality alone [4] hence it’s a consideration as a treatment option for our patient. Until recently, patients with malignant melanoma GI metastatic involvement presented with a gloomy survival rate [1]. In recent studies, survival has reached 68% [5].

## Conclusions

Metastatic involvement of the gastrointestinal tract by malignant melanoma, as well as malignant melanoma itself, needs to be borne in mind, especially in HIV-infected patients. A significant percentage of these patients will lack specific symptoms, and present with long disease-free intervals, between diagnosis of primary cutaneous MM and diagnosis of large bowel metastasis.

Diagnostic tools include CT and endoscopic exams and should be considered if symptoms arise but could also be considered in asymptomatic patients. It has recently been demonstrated that combining surgical interventions with effective systemic therapies was beneficial for this particular group of patients that are HIV-infected individuals.
